# Liver Stiffness, Patient-Reported Fatigue, and Pruritus in Primary Biliary Cholangitis and Primary Sclerosing Cholangitis: A Cross-Sectional Study

**DOI:** 10.3390/jcm15207928

**Published:** 2026-10-14

**Authors:** Victoria Abadi-Ron, Daniela Terrazas-Duque, Ana Marenco-Flores, Natalia Rojas-Amaris, Fernando Munguia, Romelia Barba Bernal, Daniela Goyes, John Esli Medina-Morales, Leandro Sierra, Behnam Saberi, Vilas Patwardhan, Alan Bonder

**Affiliations:** 1 Division of Gastroenterology, Hepatology, and Nutrition, Beth Israel Deaconess Medical Center, Harvard Medical School, 110 Francis Street Suite 8E, Boston, MA 02215, USAdterraza@bidmc.harvard.edu (D.T.-D.); fmunguia@bidmc.harvard.edu (F.M.);; 2 Oklahoma University Health Science Center, Oklahoma City, OK 73104, USA; 3 Division of Digestive Diseases, Yale School of Medicine, New Haven, CT 06520, USA; 4 Division of Gastroenterology, Washington University School of Medicine, St. Louis, MO 63110, USA; 5 Department of Medicine, Cleveland Clinic Foundation, Cleveland, OH 44195, USA

**Keywords:** primary biliary cholangitis, primary sclerosing cholangitis, cholestatic liver disease, fatigue, pruritus, liver stiffness, transient elastography, patient-reported outcomes

## Abstract

**Background:** Fatigue and pruritus are common and disabling in primary biliary cholangitis (PBC) and primary sclerosing cholangitis (PSC), but their severity is not reliably explained by standard biochemical markers. Whether liver stiffness measured by transient elastography reflects these symptoms remains unclear. This study examined associations between liver stiffness and fatigue and pruritus in PBC and PSC, and between routine biochemical measures and symptom burden. **Methods:** This cross-sectional analysis included 107 patients (56 PBC, 51 PSC) from a prospective autoimmune liver disease registry. Fatigue was assessed with the CLDQ-29 fatigue domain and pruritus with the CLDQ-29 pruritus item or the itch visual analog scale. Multivariable linear and ordered logistic regression examined associations between liver stiffness and fatigue and pruritus, adjusted for disease type, age, and sex. Spearman correlations assessed associations between liver stiffness and clinical and laboratory variables, with Benjamini–Hochberg correction. Receiver operating characteristic analysis assessed discrimination. **Results:** Patients with PBC were older (59 vs. 51 years; *p* < 0.001) and more often female (96.4% vs. 56.9%; *p* < 0.001) than those with PSC. Liver stiffness (6.0 vs. 6.8 kPa; *p* = 0.101), fatigue scores, pruritus scores, and itch VAS were similar between groups. Liver stiffness was not associated with fatigue (β = 0.005; *p* = 0.835) or pruritus (OR = 0.97; *p* = 0.190) after adjustment; only age was associated with fatigue (β = 0.021; *p* = 0.020). Among laboratory measures, only ALP remained correlated with liver stiffness after correction (ρ = 0.309; adjusted *p* = 0.027). In PSC alone, higher liver stiffness was associated with worse pruritus (ρ = −0.37; *p* = 0.009), a finding not seen in PBC. Liver stiffness alone showed limited discrimination for fatigue (AUC 0.46) and pruritus (AUC 0.56); a model with disease type, age, and sex improved discrimination modestly for fatigue (AUC 0.60) but not for pruritus (AUC 0.57). **Conclusions:** Liver stiffness was not associated with fatigue or pruritus in the overall cohort of patients with PBC and PSC, and a possible association with pruritus in PSC needs confirmation. Patient-reported outcomes should be assessed alongside biochemical and elastographic measures, and how they relate to objective measures of disease remains an open question.

## 1. Introduction

Primary biliary cholangitis (PBC) and primary sclerosing cholangitis (PSC) are chronic autoimmune liver diseases characterized by cholestasis, but differ substantially in clinical course and management [1,2,3,4]. Ursodeoxycholic acid (UDCA) remains the standard first-line therapy for PBC, with second-line agents available for the 30–40% of patients who do not achieve an adequate biochemical response, while no therapy has been proven to slow disease progression in PSC, where management focuses on surveillance, treatment of complications, and timely referral for liver transplantation [5,6,7,8,9]. Despite having different management and treatment algorithms, fatigue and pruritus are frequently described among the most disabling symptoms, and have a major impact on quality of life [10,11,12]. A prospective analysis of 1267 patients found fatigue to be frequent in both PBC and PSC, particularly among women [13]. Notably, the severity of these symptoms is not reliably explained by disease stage or standard biochemical markers of cholestasis, particularly in PBC [14,15].

Liver stiffness measurement by vibration-controlled transient elastography (VCTE) is a simple, non-invasive tool used to stage liver fibrosis in autoimmune liver diseases, including PBC and PSC [16]. Prior work linking elastography to disease outcomes in PBC and PSC has largely focused on clinical course and prognosis rather than patient-reported outcomes [17,18]. Studies of fatigue and pruritus in these diseases have typically examined biochemical and clinical correlates, such as ALP, GGT, and cirrhosis [13]. To our knowledge, no study has evaluated the elastography–symptom relationship for both fatigue and pruritus, in both PBC and PSC, within the same cohort.

In this study, the primary aim was to examine associations between liver stiffness measured by transient elastography and fatigue and pruritus in PBC and PSC. Secondary analyses evaluated associations between routine biochemical measures and symptom burden.

## 2. Materials and Methods

### 2.1. Study Design and Population

This cross-sectional study used data from the prospective Autoimmune Liver Disease Registry at Beth Israel Deaconess Medical Center (BIDMC), which enrolled patients from January 2018 through November 2025. Participants completed questionnaires during routine clinic visits. We retrieved clinical information, laboratory results, and transient elastography measurements from the study REDCap database.

Adult patients with PBC or PSC were eligible. Diagnoses were based on accepted clinical practice guidelines [19,20] and verified by chart review. Patients with overlap syndromes, including those whose diagnosis evolved during follow-up, and those with other autoimmune liver diseases were not included. This allowed the PBC and PSC groups to be examined separately.

**Figure 1:**
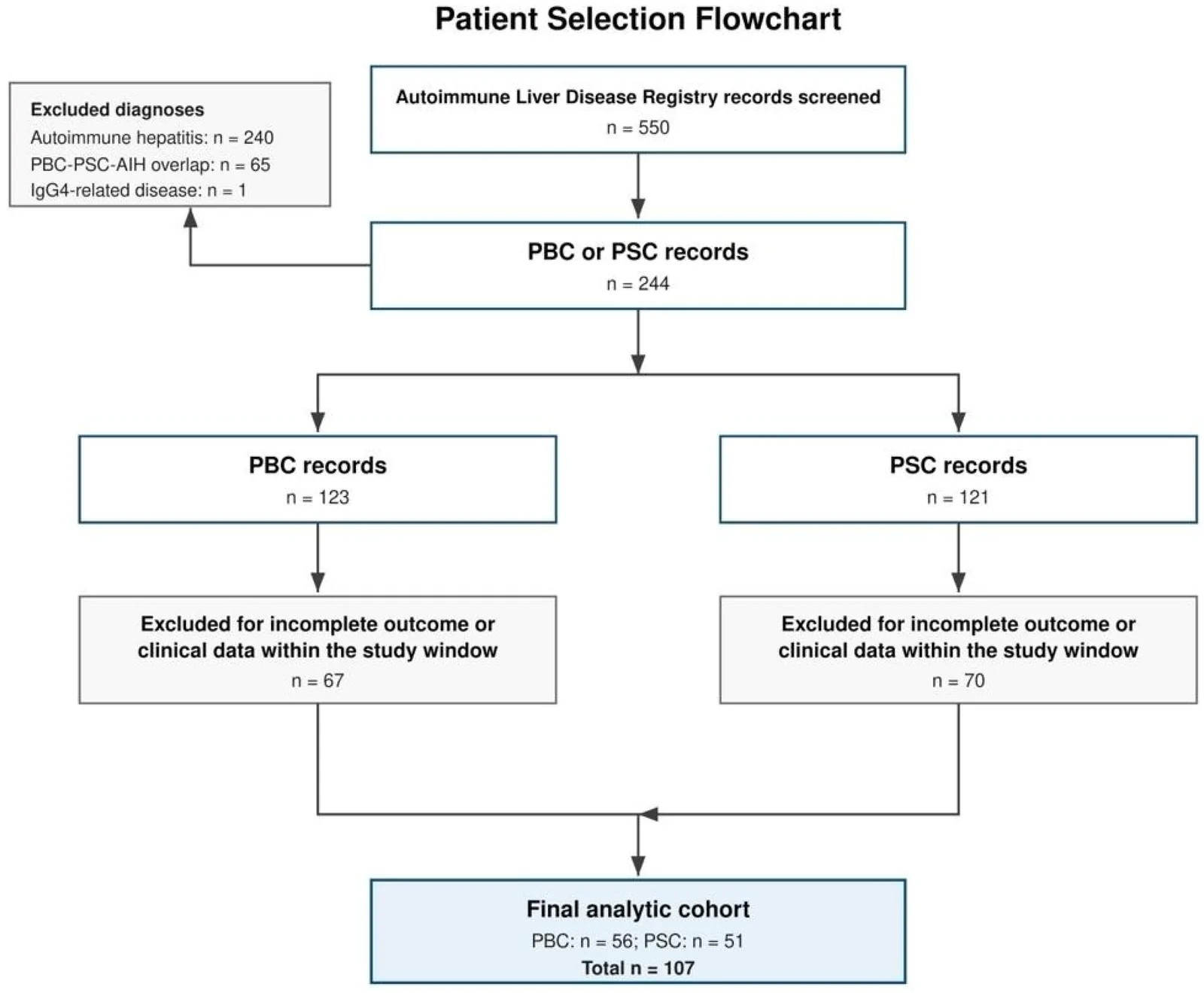
Flowchart of Patient Selection. AIH, autoimmune hepatitis; PBC, primary biliary cholangitis; PSC, primary sclerosing cholangitis; IgG4, immunoglobulin G4.

The enrollment visit served as the index visit. Participants needed complete Chronic Liver Disease Questionnaire (CLDQ)-29 responses for the fatigue score and pruritus assessment, as well as laboratory values and a valid transient elastography result obtained within six months of enrollment. Patients meeting these criteria were included in the analytic cohort. Figure 1 shows the flowchart for patient selection.

### 2.2. Patient-Reported Outcomes

The CLDQ-29 was administered at enrollment by clinicians or research staff. It assesses symptoms during the prior 2 weeks on a 1–7 scale. Fatigue-domain scores were calculated as the mean of the relevant items, with higher scores indicating better health-related quality of life.

Pruritus was assessed using either the CLDQ-29 pruritus item or the 0–10 itch visual analog scale (VAS), depending on the available response. A positive pruritus variable was defined by reported itching on either measure, allowing inclusion of patients who completed only one pruritus assessment. Higher VAS scores indicated worse itching, whereas lower CLDQ-29 pruritus-item scores indicated greater symptom burden.

### 2.3. Clinical Data, Study Variables, and Outcomes

Baseline variables included age, sex, race and ethnicity, body mass index (BMI), disease duration, alcohol use, smoking history, cirrhosis, portal hypertension-related findings, inflammatory bowel disease (IBD), sleep difficulty, depression, anxiety, and medication use. Cirrhosis was based on documented findings from abdominal ultrasound (US) or magnetic resonance imaging (MRI). For patients with PSC, the presence of IBD was recorded when available.

Laboratory measures included aspartate aminotransferase (AST), alanine aminotransferase (ALT), alkaline phosphatase (ALP), total bilirubin, albumin, platelet count, gamma-glutamyl transferase (GGT), international normalized ratio (INR), creatinine, sodium, white blood cell count (WBC), vitamin D level, Model for End-Stage Liver Disease (MELD) score, and Child-Pugh score when available. Medication variables included ursodeoxycholic acid (UDCA), fibrates, vitamin D supplementation, and pruritus-directed therapies.

Liver stiffness was measured by vibration-controlled transient elastography and reported in kilopascals (kPa). When clinically indicated, examinations were performed by one of two trained technicians. Technicians knew the clinical indication but did not have access to registry questionnaire responses. Patients fasted for at least 3 h before testing. Measurements were obtained from standardized intercostal spaces to identify the lowest valid liver stiffness value. Each examination included at least 10 valid measurements. Reliable examinations required an interquartile range-to-median ratio of 30% or less. Propagation maps were reviewed for parallel shear-wave margins and fewer than two incorrect shear-wave maps caused by rib echoes [21].

The primary outcome was to examine the association between liver stiffness and fatigue or pruritus. Secondary analyses evaluated the relationships of routine biochemical measures with these PRO and with liver stiffness.

### 2.4. Statistical Analysis

Baseline characteristics are summarized by disease phenotype. Continuous variables are presented as median and interquartile range (IQR), and categorical variables as number and percentage. PBC and PSC were compared using the Mann–Whitney U test for continuous variables and Fisher exact test for categorical variables. No formal sample size calculation was performed because the analysis included all eligible registry participants with data available for the cross-sectional study. The sample therefore reflects the available clinical registry cohort during the study period.

Multivariable linear regression was used to examine the association between liver stiffness and the CLDQ-29 fatigue-domain score. Ordered logistic regression was used for the CLDQ-29 pruritus item. Both models included liver stiffness, disease phenotype, age, and sex. These covariates were selected based on their clinical relevance and consistent availability across the PBC and PSC groups. Other clinical variables were not included as adjustment covariates because they were disease-specific, incompletely available, or evaluated separately as secondary clinical measures. The model was limited to clinically relevant covariates to avoid overfitting given the sample size. Linear-regression results are reported as regression coefficients (β) with 95% confidence intervals (CIs), and ordered logistic-regression results as odds ratios (ORs) with 95% CIs. Adjusted R^2^ and McFadden pseudo-R^2^ were reported for the fatigue and pruritus models, respectively.

Spearman rank correlations assessed unadjusted associations between liver stiffness and clinical variables, laboratory measures, fatigue, pruritus, and itch visual analog scale (VAS) scores in the overall cohort. Correlations between liver stiffness and fatigue or pruritus were also examined separately in PBC and PSC. For the overall correlation analysis, *p*-values were adjusted using the Benjamini–Hochberg method.

Receiver operating characteristic (ROC) analyses assessed the ability of liver stiffness alone and the multivariable model to discriminate fatigue impairment and pruritus burden. For the ROC analyses, fatigue impairment was operationally defined as a CLDQ-29 fatigue-domain score < 5, based on previous work using a score of 5 to distinguish lower from higher health-related quality of life and on the interpretation of the CLDQ response scale [22,23]. Pruritus burden was defined as an itch VAS score ≥ 4 on the 0–10 scale, consistent with previously proposed thresholds for at least moderate pruritus [24]. The multivariable model included liver stiffness, disease phenotype, age, and sex. Areas under the ROC curve (AUCs) are reported with 95% CIs.

Analyses used complete observations for each comparison; sample sizes therefore varied according to data availability. No imputation was performed. Analyses were conducted in Stata version 19.5 (StataCorp LLC, College Station, TX, USA). Python version 3.11 was used for data visualization. All tests were two-sided, with *p* < 0.05 considered statistically significant.

### 2.5. Ethical Considerations

The study was conducted in accordance with the Declaration of Helsinki and the principles of Good Clinical Practice. The BIDMC Institutional Review Board (IRB) approved the study under protocol 2018P00019, with approval valid through 9 December 2026. All participants provided written informed consent.

## 3. Results

### 3.1. Baseline Characteristics of the Study Cohort

The cohort included 107 patients, 56 with PBC and 51 with PSC (Table 1). Patients with PBC were older than those with PSC (median age, 59 vs. 51 years; *p* < 0.001) and were more often female (96.4% vs. 56.9%; *p* < 0.001). IBD was present in 52.9% of patients with PSC and in none with PBC (*p* < 0.001). Anxiety symptoms were also more common in PSC (33.3% vs. 10.7%; *p* = 0.005). Liver stiffness was similar between groups (6.0 vs. 6.8 kPa; *p* = 0.101). Fatigue scores, pruritus scores, and itch VAS were also similar between PBC and PSC, with no significant differences observed.

### 3.2. Factors Associated with Fatigue and Pruritus

In the multivariable analysis, liver stiffness was not associated with fatigue after adjustment for disease type, age, and sex (β = 0.005, 95% CI −0.038 to 0.047; *p* = 0.835) (Table 2). Age was associated with fatigue, with higher scores observed in older patients (β = 0.021, 95% CI 0.003–0.039; *p* = 0.020). Disease type and sex were not associated with fatigue. For pruritus, liver stiffness was also not associated with greater symptom severity (OR = 0.97, 95% CI 0.92–1.02; *p* = 0.190), and disease type, age, and sex were not significantly associated.

### 3.3. Associations Between Liver Stiffness, Clinical Variables, and PRO

In the overall cohort, ALP was the only variable that remained significantly associated with liver stiffness after Benjamini–Hochberg correction (ρ = 0.309, adjusted *p* = 0.027). Albumin and Child-Pugh score were related to liver stiffness in the unadjusted analysis, although these findings did not persist after correction. No clear correlation was seen for bilirubin, AST, ALT, MELD score, platelet count, BMI, or vitamin D level. Fatigue and pruritus also showed no meaningful relationship with liver stiffness when all patients were considered together (Table 3).

**Figure 2:**
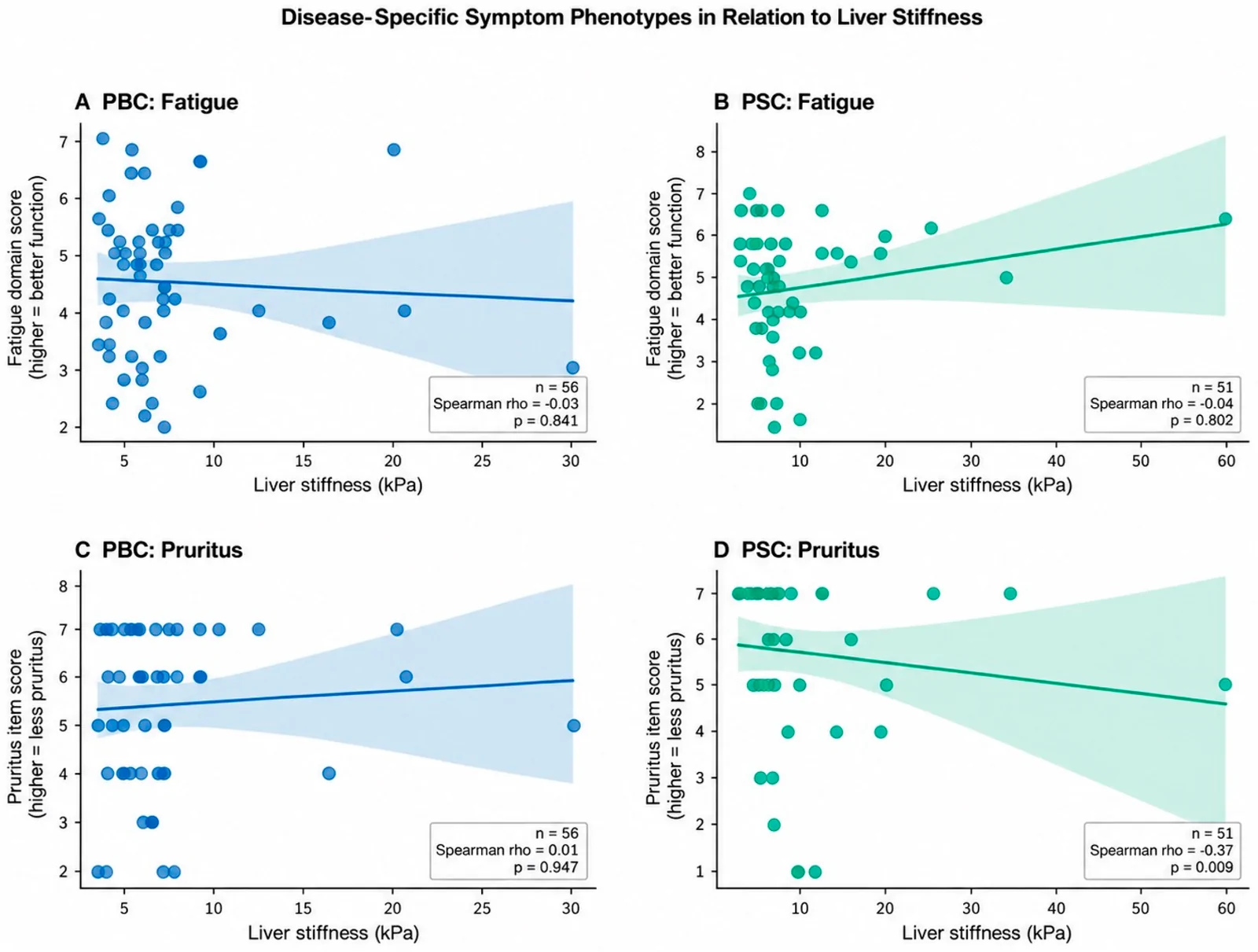
Disease-specific symptom phenotypes in relation to liver stiffness. Points represent individual patients. Solid lines show disease-specific linear fitted associations, and shaded regions indicate approximate 95% confidence bands. Fatigue scores are higher with better function, whereas lower pruritus-item scores indicate greater pruritus. Correlations are Spearman rank correlations based on complete observations.

Figure 2 shows the disease-specific relationships between liver stiffness and PROs. Fatigue was not correlated with liver stiffness in either PBC or PSC, and pruritus was also unrelated to liver stiffness in PBC. In PSC, however, higher liver stiffness was associated with lower pruritus-item scores (ρ = −0.37, *p* = 0.009). Because lower scores indicate worse pruritus, this corresponded to greater pruritus severity at higher liver stiffness values. Figure 2 also shows substantial variation in symptom scores across the range of liver stiffness, particularly for fatigue.

### 3.4. Discriminatory Performance of Liver Stiffness for Fatigue and Pruritus

**Figure 3:**
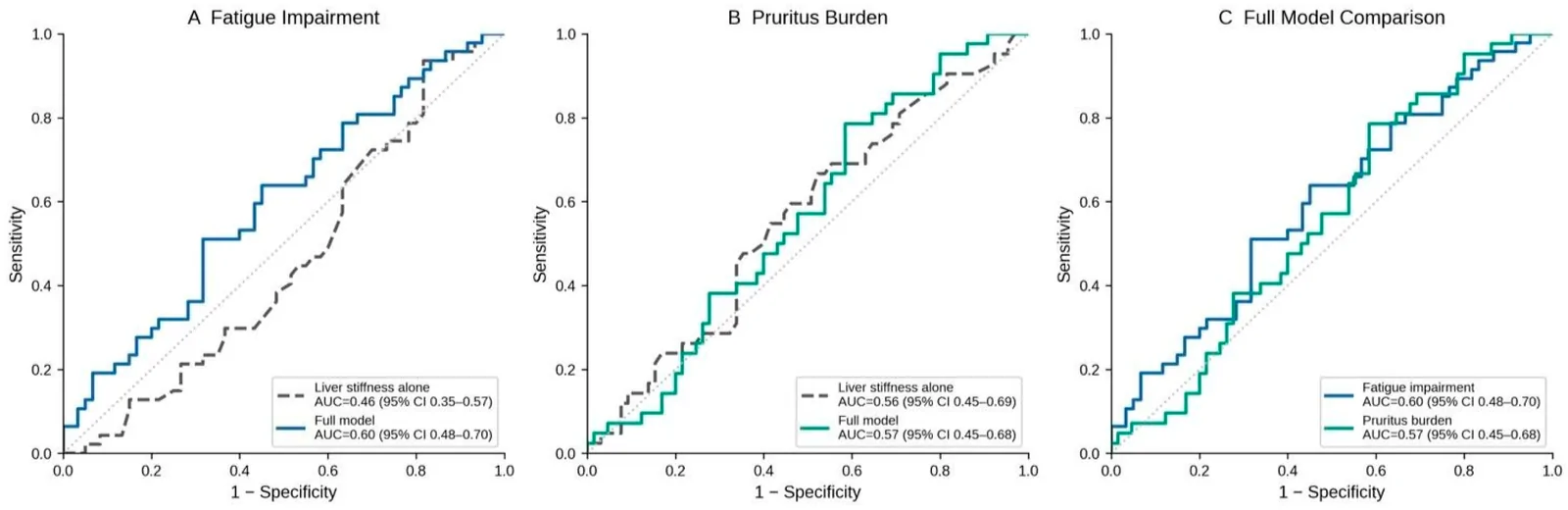
Receiver operating characteristic curves for liver stiffness and multivariable models predicting fatigue impairment and pruritus burden. ROC curves show sensitivity versus 1-specificity. Dashed gray lines represent liver stiffness alone. Solid blue and green lines represent the multivariable models for fatigue impairment and pruritus burden, respectively. AUC values are reported with 95% confidence intervals.

Figure 3 shows the ROC curve analyses for fatigue and pruritus. Liver stiffness alone had little discriminatory value for either outcome, with estimates of 0.46 (95% CI 0.35–0.57) for fatigue and 0.56 (95% CI 0.45–0.69) for pruritus. The full clinical model improved discrimination for fatigue to 0.60 (95% CI 0.48–0.70), but the estimate for pruritus remained essentially unchanged at 0.57 (95% CI 0.45–0.68). Overall, the models showed limited ability to distinguish patients according to fatigue or pruritus burden.

## 4. Discussion

In this cross-sectional analysis of patients with PBC and PSC, we found that liver stiffness was not associated with fatigue or pruritus after adjustment for disease type, age, and sex. Fatigue was also unrelated to liver stiffness within each disease, and so was pruritus in PBC. Interestingly, in PSC alone, higher liver stiffness was associated with worse pruritus in an unadjusted analysis. Among the laboratory measures, only ALP correlated with liver stiffness after correction.

Previous reports are consistent with these results, suggesting that symptoms in PBC and PSC do not clearly correlate with disease stage, which may be why they were not related to liver stiffness. In PBC, symptom burden shows no noteworthy correlation with the stage of liver disease [15]. In PSC, a recent study found no association between transient elastography and quality of life [25], although an Australian cohort linked patient-reported outcomes to disease severity, mainly decompensated cirrhosis and cholangitis [26]. Cirrhosis and advanced liver disease have been associated with worse quality of life and fatigue [27], but liver stiffness was low in our cohort and cirrhosis was uncommon (Table 1). ALP was the only laboratory measure associated with liver stiffness.

Fatigue in cholestatic disease is multifactorial, and its pathophysiology remains largely unknown [14,27]. It appears to rely on factors that stiffness does not capture. Kruk et al. linked it to ALP, GGT, and cirrhosis [13], and hypothyroidism, anemia, and depression are recognized contributors in PBC [15]. In our cohort, age was the only variable associated with fatigue. Other recognized contributors, such as IBD, anxiety symptoms, and sleep difficulty, were present in our patients (Table 1) but were not included in the models, and hemoglobin, iron, vitamin B12, folate, thyroid function, and extrahepatic autoimmune diseases were not available. Sleep disorders have been associated with greater fatigue in PBC [28].

Regarding pruritus, in PBC it was unrelated to liver stiffness, consistent with reports that pruritus severity does not always correlate with symptoms in this disease [29], and to our knowledge this relationship has not been examined before. In PSC, higher liver stiffness was associated with worse pruritus in an unadjusted, exploratory analysis; a similar pattern was reported by Hussain et al., who found greater itch intensity in patients with PSC and higher transient elastography values [30]. Pruritus also has causes that stiffness does not measure: bile acids, lysophosphatidic acid, autotaxin, and the bile acid receptor MRGPRX4 are thought to contribute, and IL-31 levels correlate with itch scores in both PSC and PBC [31,32,33].

Overall, liver stiffness did not reflect patient-reported symptom burden in our cohort, although the possible link with pruritus in PSC warrants confirmation. Patient-reported outcomes such as the CLDQ and Itch VAS should therefore be collected alongside biochemical and elastographic measures [34,35].

This study has several limitations. First, the cross-sectional design does not allow conclusions regarding causality. Second, it was a single-center study, and no formal sample size calculation was performed because all eligible registry participants were included. Third, our models adjusted only for disease type, age, and sex. Other potential confounders, including IBD, sleep difficulty, depression, and anxiety, were not included as covariates. Pruritus-directed medication and psychiatric conditions were also excluded, as too few patients had these to model reliably. Residual confounding is possible from IBD activity, thyroid dysfunction, other autoimmune or systemic disorders, anemia, and nutritional deficiencies, since hemoglobin, iron, vitamin B12, folate, thyroid function, and extrahepatic autoimmune diseases were not among the study variables. Fourth, the disease-specific correlations, including the PSC pruritus finding, are unadjusted for multiple comparisons, and the proportional odds assumption was not formally tested. Finally, questionnaires were completed at enrollment, whereas elastography and laboratory values were allowed within six months, so assessments were not simultaneous, and elastography was performed by technicians who knew the clinical indication, although reliability criteria were applied.

Future studies should be longitudinal and multicenter, with a common time window for elastography, laboratory tests, and questionnaires, and should record hemoglobin, iron, vitamin B12, folate, thyroid function, and IBD activity. They should add psychological measures as in SOMA.LIV [36], the CLDQ-PBC [37], and CLDQ-PSC [38], and IL-31 [33] as a pruritogen biomarker. New study endpoints are needed to correlate patient-reported outcomes with objective measures of disease.

## 5. Conclusions

In this cross-sectional study, liver stiffness was not associated with fatigue or pruritus in the overall cohort of patients with PBC and PSC, and a possible association with pruritus in PSC needs confirmation. These findings describe associations and do not establish causal relationships. Patient-reported outcomes should be assessed alongside biochemical and elastographic measures, and how they relate to objective measures of disease remains a major open question that requires new study endpoints.

## Figures and Tables

**Table 1:** Baseline characteristics of the study population by disease.

Variable	PBC (n = 56)	PSC (n = 51)	*p*-Value
**Demographics**			
Age (years), median (IQR)	59 (51.8–67.0)	51.0 (37.5–58.5)	<0.001
Female sex, n (%)	54 (96.4)	29 (56.9)	<0.001
BMI (kg/m^2^), median (IQR)	28.1 (24.6–32.0)	27.6 (22.9–33.5)	0.913
Disease duration (months), median (IQR)	60.4 (23.7–138.6)	97.1 (17.4–164.6)	0.653
Race/Ethnicity, n (%)			0.061
White	49 (87.5)	42 (82.4)	
Black/AA	0	6 (11.8)	
Hispanic	5 (8.9)	1 (2.0)	
Asian	1 (1.8)	1 (2.0)	
Other, mixed	1 (1.8)	1 (2.0)	
Any alcohol use, n (%)	25 (44.6)	20 (39.2)	0.695
Ever smoker, n (%)	16 (28.6)	6 (11.8)	0.054
Current smoker, n (%)	4 (7.1)	0	0.120
IBD, n (%)	0	27 (52.9)	<0.001
**Clinical characteristics**			
Cirrhosis, n (%)	5 (8.9)	2 (3.9)	0.441
Splenomegaly, n (%)	3 (5.4)	2 (3.9)	1.000
Ascites, n (%)	0	3 (5.9)	0.105
Esophageal varices, n (%)	1 (1.8)	0	1.000
Child-Pugh score, median (IQR)	5(4.0–5.0)	5.0 (4.0–5.8)	0.494
MELD score, median (IQR)	6.9 (6.0–7.8)	8.0 (7.0–9.9)	0.479
Sleep difficulty, n (%)	15 (26.8)	13 (25.5)	1.000
Depression symptoms, n (%)	4 (7.1)	8 (15.7)	0.223
Anxiety symptoms, n (%)	6 (10.7)	17 (33.3)	0.005
Psychiatric condition, n (%)	1 (1.8)	0	1.000
Liver stiffness (kPa), median (IQR)	6 (4.8–7.2)	6.8 (5.2–9.9)	0.101
**Baseline laboratory values**			
AST (IU/L), median (IQR)	25.5 (21.0–34.0)	31.5 (22.0–41.5)	0.106
ALT (IU/L), median (IQR)	25.0 (17.5–35.5)	31.0 (22.8–47.0)	0.040
ALP (IU/L), median (IQR)	129.0 (101.0–177.0)	123.0 (85.2–211.2)	0.916
Total bilirubin (mg/dL), median (IQR)	0.4 (0.3–0.6)	0.5 (0.4–0.7)	0.130
Albumin (g/dL), median (IQR)	4.4 (4.2–4.6)	4.5 (4.2–4.7)	0.245
Platelets (k/µL), median (IQR)	255 (207.0–296.0)	262.0 (199.0–301.8)	0.757
GGT (IU/L), median (IQR)	86 (39.8–128.0)	82.0 (31.0–124.0)	0.811
INR, median (IQR)	1.0 (1.0–1.1)	1.0 (1.0–1.1)	0.069
Creatinine (mg/dL), median (IQR)	0.8 (0.7–0.9)	0.8 (0.7–1.0)	0.082
Sodium (mEq/L), median (IQR)	140 (138.0–142.0)	140.0 (136.8–142.0)	0.459
WBC (k/µL), median (IQR)	6.5 (5.5–7.4)	7.0 (6.0–7.9)	0.226
Vitamin D level (ng/mL), median (IQR)	35 (28.0–43.0)	37.5 (28.8–45.8)	0.466
Vitamin D deficient (<20 ng/mL), n (%)	4 (7.1)	2 (3.9)	0.681
Vitamin D insufficient (20–30 ng/mL), n (%)	10 (17.9)	5 (9.8)	0.274
Vitamin D supplement, n (%)	25 (44.6)	18 (35.3)	0.430
**Medications**			
UDCA, n (%)	52 (92.9)	38 (74.5)	0.016
Fibrates, n (%)	2 (3.6)	0	0.496
Any pruritus medication, n (%)	6 (10.7)	2 (3.9)	0.275
Cholestyramine	0	2 (3.9)	0.225
Rifampin	1 (1.8)	0	1.000
Hydroxyzine	1 (1.8)	0	1.000
Sertraline	1 (1.8)	0	1.000
Colchicine	1 (1.8)	0	1.000
Other than specified	2 (3.6)	0	0.496
**PROs**			
Fatigue domain, median (IQR)	4.5 (3.5–5.4)	5.0 (4.0–5.8)	0.165
Pruritus item, median (IQR)	6 (4.0–7.0)	7.0 (5.0–7.0)	0.099
Itch VAS (0–10), median (IQR)	1.5 (0.0–3.0)	1.0 (0.0–2.0)	0.213

PBC, primary biliary cholangitis; PSC, primary sclerosing cholangitis; IQR, interquartile range; BMI, body mass index; AA, African American; IBD, inflammatory bowel disease; VAS, visual analog scale; MELD, Model for End-Stage Liver Disease; AST, aspartate aminotransferase; ALT, alanine aminotransferase; ALP, alkaline phosphatase; GGT, gamma-glutamyl transferase; INR, international normalized ratio; WBC, white blood cell count; UDCA, ursodeoxycholic acid; PROs, patient-reported outcomes; CLDQ, Chronic Liver Disease Questionnaire. CLDQ scores range from 1 to 7, with higher scores indicating better health-related quality of life and lower symptom burden. Continuous variables are presented as median (IQR), and categorical variables as n (%). A two-sided *p*-value < 0.05 was considered statistically significant.

**Table 2:** Multivariable analysis of factors associated with fatigue and pruritus in PBC and PSC.

Variable	Fatigue β (95% CI)	Fatigue *p*	Pruritus OR (95% CI)	Pruritus *p*
Liver stiffness (kPa)	0.005 (−0.038–0.047)	0.835	0.97 (0.92–1.02)	0.190
Disease (PSC vs. PBC)	0.346 (−0.327–1.019)	0.314	1.95 (0.80–4.76)	0.142
Age (years)	0.021 (0.003–0.039)	0.020	1.01 (0.98–1.04)	0.368
Female sex	−0.362 (−1.074–0.351)	0.320	0.69 (0.23–2.06)	0.508
Adj. R^2^/McFadden pseudo-R^2^	0.030		0.015	

PBC, primary biliary cholangitis; PSC, primary sclerosing cholangitis; β, regression coefficient; CI, confidence interval; OR, odds ratio; R^2^, coefficient of determination. Fatigue was analyzed using linear regression and pruritus using ordered logistic regression. Models were adjusted for disease type, age, and sex.

**Table 3:** Spearman Correlations with Liver Stiffness.

Variable	ρ	*p* Raw	n	*p* Adj (BH)
ALP	0.309	0.002	107	**0.027**
Albumin	−0.258	0.015	107	0.138
Child-Pugh score	0.205	0.035	107	0.212
Disease duration	0.187	0.054	107	0.221
AST	0.177	0.075	107	0.224
MELD score	0.162	0.261	107	0.427
Pruritus item	−0.157	0.111	107	0.286
Age	0.146	0.135	107	0.303
BMI	0.137	0.160	107	0.319
Total bilirubin	0.132	0.192	99	0.346
GGT	0.302	0.061	39	0.221
Itch VAS	0.076	0.439	107	0.643
ALT	−0.015	0.881	102	0.937
Platelets	−0.009	0.933	107	0.937
INR	−0.009	0.937	82	0.937
Fatigue domain	−0.018	0.858	107	0.937
Vitamin D level	−0.033	0.805	59	0.937

ALP, alkaline phosphatase; AST, aspartate aminotransferase; ALT, alanine aminotransferase; BMI, body mass index; GGT, gamma-glutamyl transferase; INR, international normalized ratio; MELD, Model for End-Stage Liver Disease; VAS, visual analog scale; BH, Benjamini–Hochberg. ρ, Spearman correlation coefficient. Bold indicates BH-adjusted *p* < 0.05.

## Data Availability

The data presented in this study are available on request from the corresponding author due to privacy restrictions.

## Abbreviations

The following abbreviations are used in this manuscript:

AILD	autoimmune and cholestatic liver disease
ALP	alkaline phosphatase
aOR	adjusted odds ratio
BIDMC	Beth Israel Deaconess Medical Center
BMI	body mass index
CI	confidence interval
CLDQ	Chronic Liver Disease Questionnaire
IBD	inflammatory bowel disease
IQR	interquartile range
IRB	Institutional Review Board
kPa	kilopascals
MRGPRX4	Mas-related G protein-coupled receptor X4
PBC	primary biliary cholangitis
PRO	patient-reported outcome
PSC	primary sclerosing cholangitis
UDCA	ursodeoxycholic acid
VAS	visual analog scale
ρ	Spearman rank correlation coefficient
